# Pathway Analysis of Expression Data: Deciphering Functional Building Blocks of Complex Diseases

**DOI:** 10.1371/journal.pcbi.1002053

**Published:** 2011-05-26

**Authors:** Frank Emmert-Streib, Galina V. Glazko

**Affiliations:** 1 Computational Biology and Machine Learning Lab, Center for Cancer Research and Cell Biology, School of Medicine, Dentistry and Biomedical Sciences, Queen's University Belfast, Belfast, United Kingdom; 2 Division of Biomedical Informatics, University of Arkansas for Medical Sciences, Little Rock, Arkansas, United States of America; Whitehead Institute, United States of America

This *PLoS Computational Biology* tutorial was presented at ISMB 2010.

## Introduction

Identification of differentially expressed pathways from expression data is an important problem because it allows us to gain insights into the functional working mechanism of cells beyond the detection of differentially expressed genes. In this paper we present a brief guide to methods for the pathway analysis of expression data. Despite the vast amount of different statistical methods that have been developed so far, there is a considerable similarity among them, allowing a systematic classification and a reduction to a few null hypotheses that are effectively tested.

Systems biology aims to find emergent phenomena by the integration of heterogeneous data. In general, data integration itself is a part of any scientific inference: its elementary steps are the integration of observations (measurements) into the context of biological knowledge. However, in the case of systems biology, the scale of integration is many folds higher, resulting in a prodigious number of new computational approaches for the simultaneous analyses of heterogeneous data. In this paper we discuss one popular way of integrating biological knowledge into large-scale genome-wide measurements, namely the identification of functionally related genes (pathways) enriched or differentially expressed in gene expression data [1]. It should be noted that the approaches discussed are also applicable to the analyses of, e.g., RNA-seq, metabolomics or proteomics data and, generally, different types of biological measurements when preexisting biological knowledge is available.

In the early stages of methodological developments for gene expression data analyses, most approaches were focused on producing so-called gene lists. This is a set of individual genes called *differentially expressed* as identified by univariate test statistics (e.g., a *t*-test) [2]–[4]. Instead, more recent approaches clearly reflect systems biology's trend of data integration and interpretation [5]–[7], focusing on sets of functionally related genes (e.g., from the same signaling or metabolic pathway) rather than individual genes.

The purpose of this paper is to provide a brief guide to methods for the analysis of differentially expressed pathways or gene sets, which we simply call pathway-based methods. For this reason, we emphasize an illustration of the methods rather than their technical description. The reader is encouraged to follow the cited literature for technical details.

## Motivation for Pathway Approaches

In order to gain a deeper appreciation for the underlying concepts of methods aiming to identify differentially expressed pathways, we briefly describe their overall goal and some basic facts of molecular systems. First of all, the ultimate goal of pathway-based approaches is to connect a molecular level with a phenotype of an organism causally or at least associatively. In the case of a disease-related phenotype, this could mean that certain molecular processes are responsible for the manifestation or development of a disease [8], [9]. The difficulty in achieving this goal is not only technical, e.g., deciding which method would allow us to decipher molecular mechanisms underlying disease phenotypes. The selection of appropriate entities at the molecular level, serving as measurement variables to capture relevant information, remains an open problem as well. Despite considerable differences between many pathway-based approaches [5], their common theme is to focus on a systems level of functional components [10]–[12] of the molecular system comprising many, as opposed to individual, genes.

The analysis of pathways that are significantly differentially expressed is intuitively appealing and there are several reasons in support of this. First, by arranging genes into pathways, the dimensionality of the dataset is reduced, and as a consequence the number of statistical hypotheses that need to be tested. Second, the statement “a gene is differentially expressed between two phenotypes” has, from a biological point of view, less explanatory power compared to the statement “a pathway is differentially expressed between two phenotypes”, because genes do not function in isolation but are interconnected with each other, forming gene networks, e.g., a transcriptional regulatory, metabolic, or protein network [11], [13]. Third, frequently, genes in a list of differentially expressed genes are highly correlated, which increases the probability of a large number of false positives. Considering pathways or gene sets instead of individual genes leverages the correlation problem to some extent, because genes in a gene set frequently act in a coordinated manner together, forming a biological process, e.g., DNA repair or protein catabolic process. Recently, an alternative approach to handle the correlation among genes has been suggested by Zuber and Strimmer [14] by calculating *correlation-adjusted t-scores* (the standardized and de-correlated mean differences between two samples). However, the idea of looking for differentially expressed pathways appeared with a different reasoning in mind. Generally, it is believed that in many diseases the changes in the expression values of genes are only moderate and undetectable for individual genes. For example, while there were no differentially expressed individual genes between Type II diabetes positive and negative patients, a set of genes involved in oxidative phosphorylation was coordinately decreased in human diabetic muscle [7]. Following this work, Subramanian et al. [15] described one of the first algorithms (Gene Set Enrichment Analysis, GSEA) focusing on the expression changes of a set of genes as opposed to changes in the expression of individual genes.

## General Aspects

Before we present pathway-based approaches, we want to note that there are two general aspects that need to be addressed properly in order to ensure a sound analysis. The first is the preprocessing of the data and the second is the correction for multiple hypothesis testing. Here, it is important to realize that the preprocessing of the data and their subsequent analysis are not independent from each other, but the preprocessing and the analysis of the data need to “fit together”. Despite the fact that these two topics do not form the major focus of this paper, we present a brief discussion to assist the reader in understanding their importance.

The preprocessing of the gene expression data obtained using microarrays addresses three issues. (1) Background correction: adjusting for hybridization effects, (2) normalization: removing systematic errors and biases to allow comparisons among arrays, and (3) summarization: combining multiple probe intensities to obtain a single value for each gene. There is a rich literature devoted to this important topic that provides guidance in the selection of appropriate preprocessing procedures [16]–[18]. A gentle introduction can be found in [19]. For more discussions about various aspects of this difficult topic, the reader is referred to [20]–[22]. The second problem that needs to be addressed is the correction for multiple hypothesis testing [23]–[26]. There are various error measures that have been used to control a Type I error rate. Principally, one can distinguish them with respect to the information that they are using. For example, there are Type I error rates based on false positives (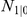) or on the false discovery proportion (FDP). Here, the false discovery proportion is 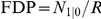 for *R*>0 and zero for *R* = 0, with *R* being the number of significant tests. In the context of microarray data for identifying differentially expressed genes, there have been extensive studies conducted providing guidance in selecting an appropriate multiple testing procedure [3], [27], [28]. However, for pathway-based approaches, this problem has received considerably less attention and is currently still under investigation. For this reason, it is advisable to investigate carefully what error rate and procedure is most appropriate for given circumstances.

## Pathway-Based Approaches

**Figure 1:**
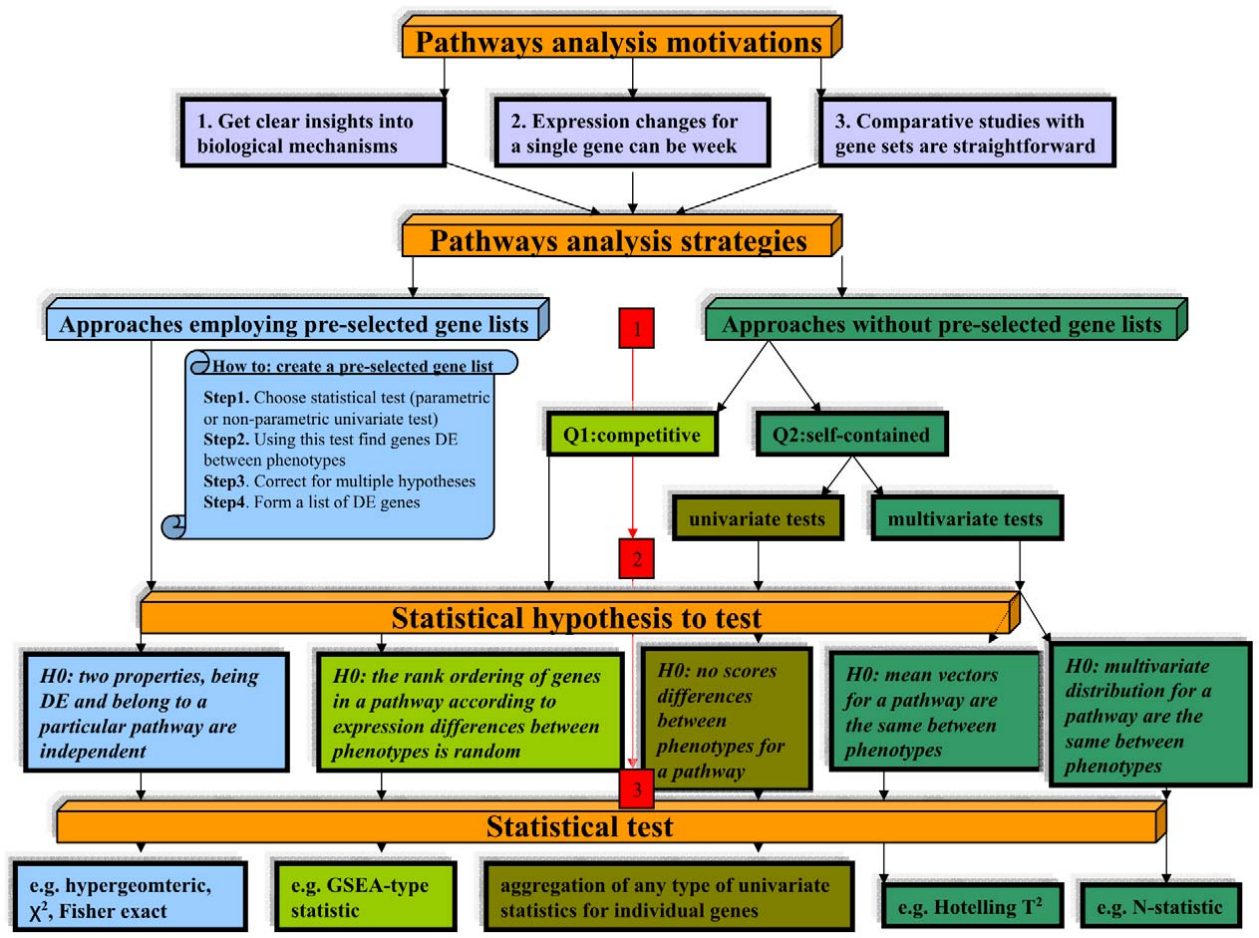
An overview of motivations and strategies, underlying statistical hypotheses and corresponding tests for pathway-analysis.

In the following, we provide an overview of different pathway-based methods. Figure 1 illustrates a general taxonomy of various pathway analysis strategies. Overall, there are three major decisions to make (indicated by the numbers in the red boxes in Figure 1): The first decision (Figure 1, red box 1) defines whether pre-selected gene lists are used in the analysis. The second decision (Figure 1, red box 2) determines the type of the null hypothesis (*H*_0_) that will be tested in the analysis. The third decision (Figure 1, red box 3) connects particular null hypotheses and statistical tests.

It appears natural that the earliest pathway-based approaches resulted from the analysis of the differential expression of individual genes (Figure 1, left column “over-representation analysis”, also called “gene lists”). The analysis of the differential expression of individual genes results in a gene list, i.e., a data sheet of genes called differentially expressed (DE) as declared by an univariate test (see Figure 1, “How to: create a pre-selected gene list”). We want to emphasize that this gene list is called a *pre-selected gene list* in the literature [29]. This is an unfortunate convention because it is easy to confuse this gene list with a gene set as defined by, e.g., the Gene Ontology (GO) database (see below). Then, instead of considering genes one by one, one can ask “Do all these genes, declared differentially expressed, have any biological function in common?” To answer this question one should know the gene sets with common biological functions. These gene sets can be defined either ad hoc as genes that are “interesting”, e.g., the set of prostate cancer-related genes, or, as is more common in this type of analysis, using functional categories, e.g., from the GO database [30]. The next step is to decide whether a set of interest, e.g., from GO, is overrepresented in the DE set. Here, overrepresented could mean that genes involved in apoptosis appear more frequently than expected by chance in the list of DE genes. Many conventional statistical tests can be applied for answering this question, e.g., Fisher's exact test (see Figure 1, Table 1, and [29], [31] for a review). However, despite its popularity and simplicity, this approach has several shortcomings. For instance, the power of this approach is entirely defined by the list of pre-selected genes. The content and the size of a gene list, in turn, is defined by the types of the univariate test statistic and multiple testing procedure chosen for selecting individual genes; see Allison et al. [32] for more discussions about the analysis of individual differentially expressed genes. Most importantly, over-representation analysis ignores all genes that were not included in the list of pre-selected genes, increasing the chances for missing a biological signal [29], [33]. The approaches without pre-selected gene lists (Figure 1, right column “Approaches without pre-selected gene lists”, and Table 1) do not have these limitations. For this reason we focus in the remainder of this paper on the latter approach.

**Table 1:** Overview of different pathway-based methods.

Principle Method	Reference	Type	Software
Over-representation analysis	Huang et al. [29]	Competitive	GOstats and http://www.geneontology.org/GO.tools.microarray.shtml
Gene set enrichment analysis	Mootha et al. [7]	Competitive	GSEABase and http://www.broad.mit.edu/gsea/
	Subramanian et al. [15]	Competitive	GSEABase and http://www.broad.mit.edu/gsea/
	Efron et al. [58]	Competitive	No
GAGE: GSEA extension	Luo et al. [59]	Competetive	GAGE
PAGE	Kim et al. [35]	Competitive	PGSEA, GAGE
Random Sets	Newton et al. [60]	Competetive	Part of CLEAN
Generalized Random Sets	Freudenberg et al. [61]	Competetive	http://GenomicsPortals.org/
Average of single-gene statistics	Tian et al. [48]	Self-contained	sigPathway
Linear Model Toolset for GSEA	Jiang et al. [49]	Self-contained	GSEAlm
SAM-GS	Dinu et al. [62]	Self-contained	http://www.ualberta.ca/~yyasui/SAM-GS/
globaltest	Goeman et al. [63]	Self-contained	globaltest
GlobalANCOVA	Hummel et al. [46]	Self-contained	GlobalAncova
Hotelling's *T* ^2^	[43]–[45]	Self-contained	PCOT2
N-statistic	Klebanov et al. [47]	Self-contained	Cramer, R package

Where available, a link to the software or the name of the Bioconductor package (http://www.bioconductor.org/help/bioc-views/release/bioc/) [57] is provided.

## Principle Differences: Null Hypothesis

One in the meanwhile classic approach that does not rely on pre-selected gene lists is GSEA [7], [15]. The simplified working mechanism of the GSEA method can be summarized as follows: (1) Rank all genes in a dataset according to their expression differences between two phenotypes. (2) For each gene set (groups of functionally linked genes from, e.g., GO) calculate an enrichment score (ES), where ES is a running sum statistic reflecting the spread of the members of a gene set among all ranked genes. From this select the maximum enrichment score (MES). (3) Calculate the significance of the MES from the null distribution of MESs for phenotype-label randomized data.

Since the appearance of GSEA, many approaches have been suggested for the analysis of gene sets [34]–[37] and their number is still growing; see Ackermann and Strimmer [34] for a review. All these approaches aim to identify gene sets that change their expression significantly between phenotypes, where genes in a set may belong to the same biological process. The definition of gene sets can be obtained from databases like the Kyoto Encyclopedia of Genes and Genomes [38], Gene Ontology [30], GenMAPP [39], or ResNet [40]. Goeman and Bühlmann have argued [6] that the major difference between these approaches can be formulated in terms of *competitive* and *self-contained* tests. Competitive tests compare the differential expression of a gene set against the remainder of all genes, and self-contained tests answer the question whether a gene set is differentially expressed between different phenotypes. Subsequently, different null hypothesis Q1 and Q2 are tested [6] (Figure 1, right column, Q1 and Q2; and Table 1).

(Q1) Null hypothesis of competitive approaches:

The genes in a set are as often differentially expressed as the genes in the rest of the sets.

(Q2) Null hypothesis of self-contained approaches:

No genes in a set are differentially expressed.

Dinu and colleagues [41] have demonstrated that the power of competitive and self-contained tests cannot be compared objectively in simulation studies because the decision as to which test has more power depends crucially on the hypotheses (Q1 or Q2) underlying the simulation of the data, favoring the data-generating hypothesis. On the other hand, several arguments have been raised in favor of self-contained tests [6]:

1. They represent an immediate generalization of single-gene tests.
2. Their null hypothesis has a clear biological interpretation.
3. They make sense even if we consider all genes on a chip simultaneously, whereas a competitive test does not.

In summary, this means self-contained tests are easy to interpret biologically and they can be more powerful compared to competitive tests. Table 1 provides an overview of various competitive and self-contained tests, including information about the availability of software implementations. In the following we discuss self-contained tests only, and the interested reader is referred to [42] for a comparative power analysis of competitive tests.

## Differences among Self-Contained Tests

Self-contained tests can be distinguished in terms of whether they are multivariate and account for interdependencies among genes (e.g., Hotelling's *T* ^2^ test: [43]–[45]; GlobalANCOVA: [46]; N-statistic: [47]; Table 1) or disregard existing complex correlation structures in a gene set and consider gene-level statistics only (e.g., weighted sum of *t*-tests: [48]; median-based or sign-tests: [49]; Table 1). Further, for gene-level statistics, a transformation of the test statistic is frequently applied in order to account for the presence of up- and down-regulated genes in a gene set [34]. However, more importantly, for univariate and multivariate self-contained tests, the underlying statistical hypotheses are different. For example: Hotelling's *T* ^2^ tests the equality of two multivariate mean vectors, while the N-statistic tests the equality of two multivariate distributions. A combination of univariate statistics (either transformed or not) assesses whether the aggregate gene-level test score differentiates between two phenotypes [49]. We want to emphasize that due to these complementing null hypotheses, each test projects on different aspects of the data. There are many more self-contained tests available [34]; however, effectively, there appear to be barely more than three general types of underlying null hypotheses being tested [1].

In order to choose the most appropriate test, one needs to know their relative power in different settings and the different null hypotheses they test. For this reason, we presented in [1] a comparative power analysis for univariate and multivariate self-contained tests on simulated and biological data focusing on three major issues. First, not all genes in a gene set change their expression between different phenotypes. The percent of genes that are actually changing their expression in a gene set, referred to as detection call, in the way that the entire gene set is called differentially expressed, is an important, but currently unknown, characteristic of the performance of a test. Second, genes in a gene set that are functionally related to each other might exhibit a complex correlation structure [50]. Multivariate tests might have a higher power because they account for interdependences among genes considering the joint distribution of gene expression levels, in contrast to univariate tests, which test differences in the marginal distributions. The third question is an implication of the second: one might expect that because univariate and multivariate statistics test different null hypotheses, for real biological data they may result in completely different gene sets. There is a reason for concern here: for example, the application of Principal Component Analysis and gene-level tests resulted in exactly this scenario [49]. In [1] we answered the first two questions with simulated data, mimicking the stated conditions, and the third one with two biological data sets from acute lymphoblastic leukemia and NCI-60 cell lines. As a result, we found that all tests perform reasonably well in estimating the Type I error rate. Among the three parameters varied in the simulations (the magnitude of pairwise correlations among gene expressions, the number of genes changing their expression in a set, and the size of a gene set), the magnitude of pairwise correlations has the largest influence on the power of all tests. Despite the general belief that multivariate tests account for a complex interdependence structure between genes and, hence, may result in a better power compared to univariate tests, our study demonstrated that this is not true when high correlations are present. Further, we found that the performance of all tests coincides when the following three conditions hold:

1. The correlation among genes is low.
2. The number of genes in a pathway is relatively large.
3. The percent of genes changing significantly their expression (detection call) is high.

Due to the fact that for biological data these three conditions may hold only with varying degree, differences in these tests are expected. From the two univariate and three multivariate self-contained tests used in our previous study, only three of them can be considered conceptually different with respect to their underlying null hypotheses. It appears that these three null hypotheses cover the vast majority of the current universe of all self-contained tests employed until now. Due to their complementing null hypotheses, each test projects on different aspects of the data. This suggests the simultaneous usage of several tests in order to gain power compared to each of these tests individually. For technical details about pathway approaches, the reader is referred to the following recent review papers [34], [41], [51].

## Discussion and Conclusions

The analysis of pathways or gene sets differentially expressed between phenotypes has became a routine approach for the analysis of gene expression data. Despite the wealth of different methods available for such an analysis, there exist considerable similarities among them, allowing for a systematic classification and a reduction to a few null hypotheses that can be tested effectively [1], [34]. Figure 1 illustrates that at present there appear only to be five different null hypotheses behind all pathway analysis strategies. An important take-home message from this is that testing all these null hypotheses would be the most comprehensive way to highlight different aspects of the data and increase the chances of retrieving a meaningful biological signal. In addition, it would allow one to distill a strong biological signal, if present, in the intersection of the results. We expect that further developments in this field will allow for the consideration of the heterogeneity of gene expression in a gene set and also allow for the integration of additional biological information, e.g., the topology of a pathway [52] in the analysis. Another problem that deserves more attention is the overlapping among gene sets that leads to complications in the interpretation of obtained results. An *enrichment map* has been suggested as a visual interpretation guide [53], but further investigations are necessary to address the hierarchical organization among these gene sets; see also [54], [55] for further attempts in this direction. Finally, we would like to emphasize that despite the fact that in this paper we focused entirely on expression data from microarray experiments, many of the discussed methods translate to data from other technology platforms, e.g., RNA-seq [56].

We conclude with a general note of caution. Although many of the presented methods are available as easily usable software packages, we do not want to give the impression that these methods should be used in a plug-and-play manner. Quite the contrary. Each of these methods and the resulting findings need to be selected, applied, and interpreted mindfully, paying close attention to relevant statistical and domain-specific details in order to impede fallacious conclusions.
